# Editorial: Ethics and COVID-19: The bioethics of a “job well done” in public health

**DOI:** 10.3389/fmed.2022.996408

**Published:** 2022-10-17

**Authors:** Vittoradolfo Tambone, Anna De Benedictis, Jane Wathuta, José López Guzmán, Francesco De Micco

**Affiliations:** ^1^Research Unit in Bioethics and Humanities, Campus Bio-Medico University of Rome, Rome, Italy; ^2^Operative Unit of Clinical Direction, University Hospital Campus Bio-Medico Foundation, Rome, Italy; ^3^Research Unit of Nursing Science, Campus Bio-Medico University of Rome, Rome, Italy; ^4^Strathmore University Institute for Family Studies and Ethics/Strathmore Law School, Nairobi, Kenya; ^5^Department of Pharmacology and Toxicology, Faculty of Pharmacy, University of Navarra, Pamplona, Spain

**Keywords:** COVID-19, job well done, public health ethics, clinical risk management, policy and practice

The COVID-19 pandemic has tested the capacities of health care systems and raised new challenges related to ethical, medical humanity, communication, psychological, patient safety, and clinical risk management issues. In addition, the COVID-19 pandemic revealed that it is no longer possible to make medicine from medicine alone, but that every reality with which humans are confronted can have an effect on health, showing a systemic dimension of medicine (1), in which the ethics of a “job well done” is the foundation and effect of an integrated collaboration between health professionals.

The ethics of a “job well done” has, as its theoretical objective, the enhancement of the moral object of the Human Act which, in public health, provides the main content of best practice and of the care gold standard. The aim of this Research Topic is to highlight the ethical issues that emerged during the pandemic and how these were addressed according to an approach consistent with the definition of a “job well done.”

COVID-19 has shown how interactions between biological and social factors can negatively influence the prognosis and treatment of a disease, supporting the reasoning of those who consider COVID-19 not as a pandemic, but rather a syndemic phenomenon (1). A syndemic approach provides an important orientation for clinical medicine because it reveals how socio-biological interaction can affect the course of a disease. Similarly, the syndemic assessment of a biological phenomenon provides methodological support to public health to guide health policy choices (2).

For this reason, we argue that the WHO has promoted a syndemic approach for the next decade to improve the quality of healthcare and ensure patient safety. The WHO has encouraged multi-disciplinary approaches based on the implementation of protective legislative measures, health systems characterized by good governance, transparency and a no-blame culture, patient and family engagement, identification of centers of excellence in patient safety education, and training and development of multi-sectoral and multinational synergies (3).

Governments and health systems around the world have experienced unprecedented stress: globally, there have been more than 300 million confirmed cases of COVID-19, including more than 5 million deaths (4, 5).

The pandemic emergency has also raised important bio-political, bioethical, and bio-juridical questions (6), which also emerge from this collection. In particular, original papers published in the special issue address the main themes detailed below.

First, the patients' access to care in conditions of limited health resources, and the related search for appropriate criteria to determine the ceiling of care (Bhattarai et al.; D'Errico et al.) represents one of the main challenges for governments and healthcare facilities, together with the need to address ethical and legal issues of telemedicine (De Micco et al.) and new risks and benefits due to the increased use of digital tools in health care (Oliva et al.) (7, 8).

In addition, some of the most relevant questions faced during the pandemic include the safety and protection of frontline healthcare professionals (Piredda et al.; Zhao et al.) while ensuring the best possible person-centered care for all patients (De Benedictis et al.), the consideration of ethical implications of the social determinants of health (Valera et al.), and the need to include the voices of patients in research, development, and care activities (Mirpuri et al.). The international debate also focused on the need to prevent the dissemination of inaccurate information from unreliable sources, while guaranteeing freedom of expression (Bakuri et al.).

Moreover, the legal and bioethical issues of vaccination which emerged from the pandemic should be addressed from different points of view (Inoue). Some of the most debated questions concern the vaccine hesitancy phenomenon (Raballo et al.) and the ethical and legal questions of compulsory COVID-19 vaccination (Gibelli et al.). At the same time, it is necessary to reflect on people's acceptance of vaccination, with a focus on different setting and low-resource settings (Maccaro et al.), and to shed new light on questions related to vaccinations for vulnerable groups of people (Scendoni et al.).

According to UNESCO's International Bioethics Committee (IBC) and the World Commission on the Ethics of Scientific Knowledge and Technology (COMEST), the impact of the COVID-19 pandemic on public health requires a global bioethics reflection and response (9).

We believe that the most advanced vision of bioethics is that which creates, together with medicine, a true co-working relationship. This methodological perspective certainly has its roots in the choice of an ethics of the first person quite distinct from ethical proceduralism of a utilitarian type (10). Scientific action (like any human action) is first of all a *Human Act* carried out by a *subject* together with other human beings, within a specific ecosystem and with a broad political dimension.

Thanks to the theory of complexity and systemic thinking (11, 12), the concept of “medicine made only within medicine” is outdated. Nowadays, talking about public health implies being aware of the impact that nutrition, industrial production, communication, and many other areas of human action have on the health of everyone (13). For example, the Covid-19 pandemic affected the education of young people (14), and it has also changed the economy of entire countries (15), in addition to the increased risk of violence against women (16).

On the other hand, the awareness of the systemic dimension of human existence and, consequently, the decisive importance of co-working as a “job well done,” brings the model of human work back from the individualistic dimension to that of conscious cooperation (17).

The main features of an approach to work based on the bioethics of a “job well done” are the following (14):

(a) interdisciplinary co-design in relation to complexity theory and systemic thinking;
(b) realistic knowledge that always starts from experience and leads to the search for scientific truth as the basis for one's choices;
(c) maintaining the purpose of medicine by going beyond the temptation to reduce it to a “business model,” instead moving toward a “Living Company Model” capable of developing a management model that is useful for the motivational involvement of all the components involved;
(d) awareness that every medical act is a free and responsible *Human Act* with an intrinsic ethical value;
(e) recovery of the political dimension of work well done, whereby professional excellence becomes a means of serving society and the common good;
(f) capacity for radical procedural innovation and not just implementation of correct procedures;
(g) putting the person at the center of work, always starting with the best evidence available.

The ethics of a “job well done” develops and justifies specific and concrete professional characteristics to improve effectiveness and efficiency, while ensuring sustainability. The pandemic emergency poses the ancient and ever new challenge described in one of the most influential frameworks for quality assessment in healthcare put forth by the Institute of Medicine (IOM), that is, caring for patients in a safe, effective, person-centered, efficient, equitable, and timely way. This framework is aimed at avoiding injuries to patients; providing evidence-based healthcare services that respond to individual preferences, needs and values; reducing waiting times and sometimes detrimental delays; avoiding waste; and, providing the best care for all (18, 19). A new paradigm of doing medicine is the way to achieve these goals for individual and public health.

Professionals at all levels over the course of the pandemic experienced the power of interprofessional and interdisciplinary collaboration in providing the best possible care for all patients, within highly interdependent healthcare environments (20).

At the same time, new patient needs emerged and health professionals are faced with an extraordinary challenge of treating fragile patient categories, while also ensuring their safety and aspirations for the best possible treatment in a person-centered way (De Benedictis et al.). In this new scenario, public health should be guided by new drivers, including the voices of patients, frontline professionals and caregivers and their ever increasing involvement in research, development, and care activities (Mirpuri et al.) (21, 22).

For this reason, it seemed necessary to propose a special issue that would observe the same clinical reality from many different points of view. The main objective was to provide “raw material” to those who want to independently compose the “puzzle” of a more systemic proposal for the governance of COVID-19 (Figure 1), based on the “Ethics of job well done framework” (De Micco et al.). We are still learning how to deal with a pathology that has a variety of novel characteristics and we are discovering many unexpected things by observation of the evidence. We are yet to fully understand what exactly has happened and is still going on, but what is clear is that we need to take care of people “all together” in a vision that moves from a regional Public Health to a Systemic Public Health (3).

**Figure 1:**
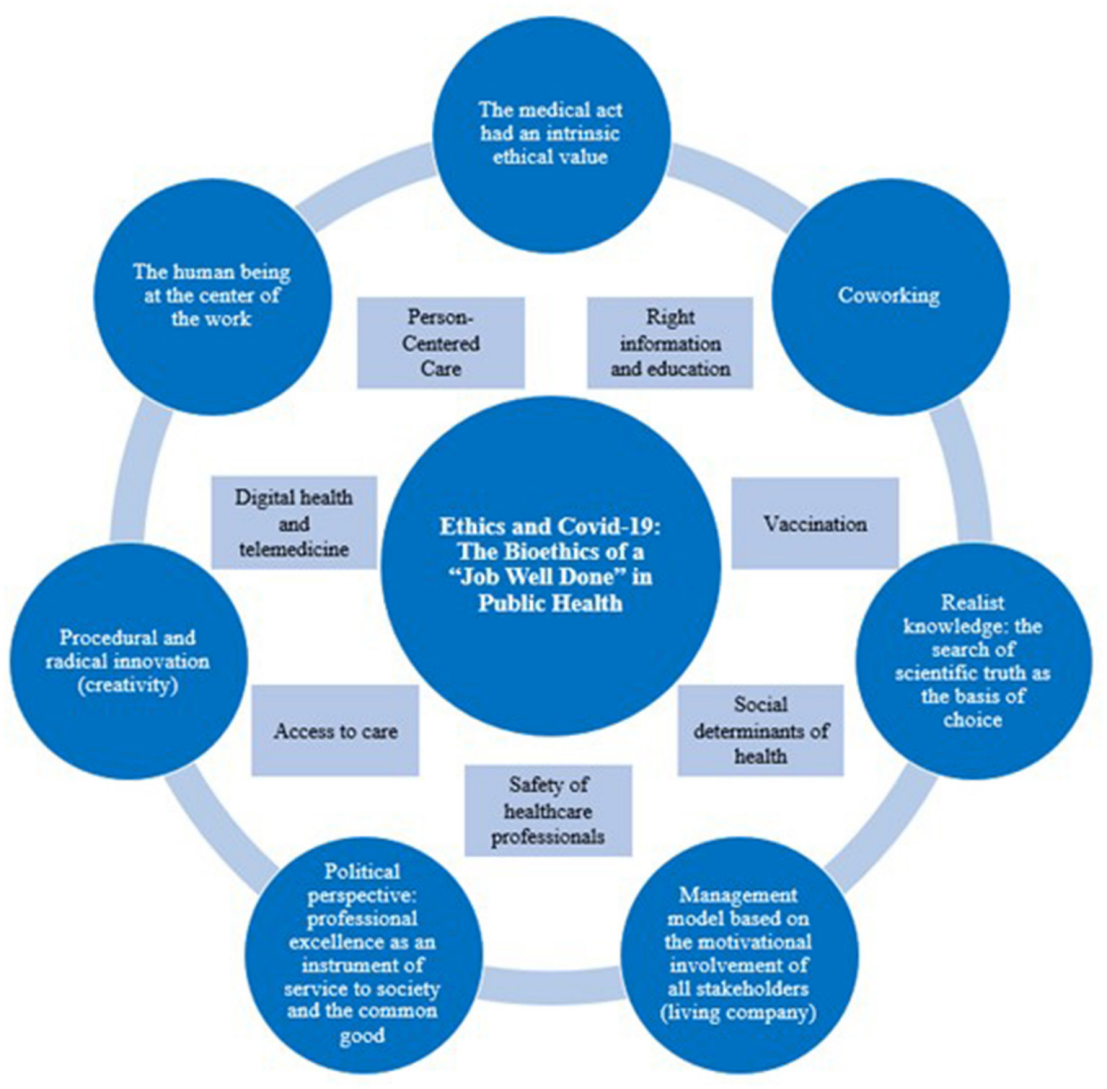
The main themes that emerged from the Research Topic with respect to the “Ethics of Job Well Done framework” (De Micco et al., modified).

## Author contributions

VT wrote the first version of the manuscript. All authors made a significant contribution to this paper and have read and approved the final version of the manuscript.

## Conflict of interest

The authors declare that the research was conducted in the absence of any commercial or financial relationships that could be construed as a potential conflict of interest.

## Publisher's note

All claims expressed in this article are solely those of the authors and do not necessarily represent those of their affiliated organizations, or those of the publisher, the editors and the reviewers. Any product that may be evaluated in this article, or claim that may be made by its manufacturer, is not guaranteed or endorsed by the publisher.
